# Editorial of Special Issue “Genetics and Molecular Pathogenesis of Non-Ischemic Cardiomyopathies”

**DOI:** 10.3390/ijms21249398

**Published:** 2020-12-10

**Authors:** Raffaella Lombardi, Suet Nee Chen

**Affiliations:** 1 Department of Advanced Biomedical Sciences, Federico II University of Naples, Via Pansini 5, 80131 Naples, Italy; 2 Department of Medicine, Division of Cardiology, University of Colorado Anschutz Medical Campus, 12700 E 19th Ave # F442, Aurora, CO 80045, USA

This editorial aims to summarize the eight scientific papers published in the Special Issue “Genetics and Molecular Pathogenesis of Non-ischemic Cardiomyopathies”.

Cardiomyopathies are primary diseases of the myocardium, and are leading causes of sudden cardiac death (SCD), particularly in athletes and the young. Diagnosis of affected individuals remains challenging, and a resolutive therapy is still lacking, as current treatments can only attenuate the symptoms but not prevent or rescue the phenotype (1). Genetics and genomics along with studies in cellular and animal models are fundamental for delineating the genetic bases of cardiomyopathies and to elucidate the pathogenesis of these diseases. This could greatly improve diagnosis and prognostic evaluation of the affected individuals as well as their family members and lead to the identification of novel targets for more effective treatments.

The papers published in this Special Issue discuss several aspects of genetics, genomics and mechanistic studies in the field of primary cardiomyopathies.

The role of genetic screening by next generation sequencing has been discussed by several papers in the Special Issue [1,2,3,4,5]. One of these papers [2] addresses the topic of applicability of genetic testing in the diagnostic work-up of athletes referred to tertiary cardiology centers for a suspicion of inherited cardiac disease. The identification of athletes at increased risk of SCD is a very complex issue; in fact, the structural, functional and electrical remodeling of the athlete’s heart often leads to electrocardiographic (ECG), echocardiographic and cardiac magnetic resonance (CMR) modifications which make the differential diagnosis between myocardial disease and the athlete’s heart a challenging task for cardiologists. In this subgroup of subjects, genetic testing may be resolutive. The paper by Barretta et al. [2] highlights the genetic heterogeneity of inherited arrhythmogenic diseases, which imposes the need for advanced sequencing methods to sequence multiple genes simultaneously to confirm the causal mutation and modifier variants. Moreover, the paper underlines the need for the right expertise to correctly interpret the genetic results in order to define causality and pathogenicity of the variants. Barretta et al. also emphasize that the maximum clinical efficacy from the genetic testing in the pre-participation screening program can be obtained only through a correct selection of the eligible athletes on the basis of phenotypic features.

A comprehensive review by Patel et al. [3] describes the complex genotype–phenotype relationship in Arrhythmogenic Cardiomyopathy (ACM), an inherited cardiomyopathy characterized by ventricular arrhythmia and an increased risk of SCD. Several genetic determinants and phenotypic manifestations have been discovered in ACM; furthermore, clinical evaluation has revealed incomplete penetrance and variable expressivity. The complexity of the genetics and phenotypic manifestations of ACM requires an integrated approach, combining data from multiple sources such as electrocardiographic, arrhythmic, morphofunctional, histopathological findings, genetics and molecular cardiology. The authors of the review describe, in detail, the genetic basis of ACM with specific genotype–phenotype associations. In particular, they focus on the genetic overlaps between ACM and other genetic cardiomyopathies characterized by an increased arrhythmic risk. Moreover, they prospect the necessity in the near future for a precision medicine-based management in ACM.

ACM incomplete penetrance and phenotypic variability suggest that the causal gene is not the only determinant of the phenotype and that other factors may modulate the disease manifestations. Among them, microRNAs (miRNAs)—small noncoding RNAs with a primary role as regulators of gene expression and cell–cell communication—have been proposed as potential players by several authors. The paper by Bueno Marinas et al. [4] published in this Special Issue focuses for the first time on the identification of specific circulating miRNAs in a large ACM cohort (unbiased by technical and biological factors). The authors identify a panel of six dysregulated miRNAs which were able to differentiate ACM patients from healthy subjects and also to distinguish patients with ongoing disease from silent family pathogenic-variant carriers. In addition, the six-miRNA panel was able to distinguish ACM patients from patients with other cardiac diseases (including channelopathies, other primary cardiomyopathies and myocarditis). Hence, the unique six-miRNA panel identified in this study exhibits a great discriminatory diagnostic power and appears to be specific for ACM, which makes it the best potential diagnostic biomarker reported to date. The implication of miRNAs in the pathogenesis and their role as biomarkers in ACM were presented by the same group in a review [5], in which the authors provide a summary of multiple studies published on this interesting topic and discuss experimental designs and methodologies on miRNA detection, highlighting evidence and flaws in each study. Moreover, the review reports the current knowledge of the implication of miRNAs in ACM pathogenesis and diagnosis. An increasing number of studies on ACM human samples, cell cultures and animal models strongly suggest that miRNAs play an important role in the disease pathogenesis; indeed, most of the miRNAs identified in these studies affect well-established ACM pathways such as the canonical Wnt/beta-catenin signaling, or the Hippo pathway. However, the findings reported in the literature remain inconclusive. The authors suggest that differences in techniques, type of specimens and analysis methods may explain the lack of reproducibility among the different studies. Hence, the authors propose the necessity for a joint effort to analyze results, compare methodologies, and formally test the robustness of miRNA associations, in order to validate miRNA as diagnostic and prognostic biomarkers in cardiomyopathies.

The effects of lifestyle and in particular the effects of sexual interactions on the cardiac function, clinical presentation and survival have been studied in a paper published in this Special Issue in an experimental mouse model of dilated cardiomyopathy (DCM) [6]. It has been estimated that worldwide there are about 64.3 million people with heart failure (HF) and that in developed countries, HF has a prevalence of 1% to 2% of the general adult population [7]. Although it has been shown that sexual activity enhances the quality of life of these patients, the safety and long-term effects of sexual activity in patients with HF are not fully known due to a paucity of experimental data. The paper by Tripathi et al. [6] tested for the first time whether lack of sexual interactions affects pathophysiological outcomes in a pre-clinical mouse model of DCM. The authors show that sex deprivation in male mice with DCM leads to accelerated decline in heart function, progressive fluid retention, higher testosterone levels and reduced survival. The findings suggest that sexual interactions may have beneficial effects in DCM, mainly mediated by changes in testosterone levels. However, how sexual activity reduces testosterone levels and the underlining molecular mechanisms remain to be discovered. The authors suggest that sexual interactions may alter cardiac transcription networks to reduce HF progression and improve survival.

The role of the extracellular matrix (ECM) and of the cytoskeleton on the pathogenesis of cardiomyopathies has never been discussed in detail. In the Special Issue, two papers published by two research groups discuss these novel topics. HF is the final stage of several cardiovascular diseases, and HF with a preserved ejection fraction (HFpEF) represents half of the cases. However, no treatment has yet been shown to reduce morbidity and mortality in patients with HFpEF. The opinion letter from Hochman-Mendez et al. [8] discusses the main determinants of the elevated passive myocardial stiffness observed in HFpEF, which are: increased ECM stiffness due to increased deposition of collagen and higher cardiomyocyte stiffness due to changes in the sarcomeric protein titin. Titin is a large sarcomeric protein that works as a molecular spring connecting the z-lines to myosin in the cardiomyocyte. Titin’s elastic properties determine the passive mechanical properties of cardiomyocytes and can be modulated by two main mechanisms: phosphorylation and isoform-shift. Cardiomyocytes are surrounded by the basement membrane, a thin, highly specialized layer of ECM mainly composed by laminin. Costameres, protein-structures composed of integrins and dystroglycans, connect and transmit molecular signals and mechanical forces between the basal lamina and the cardiomyocytes in a well-orchestrated network. Hence, the authors suggest that manipulating basement membrane proteins, specifically laminin, could modify cardiomyocyte stiffness. Indeed, based on their own preliminary in vitro data, they propose polylaminin, a biomimetic polymer of laminin, as a promising approach for manipulating the titin isoform shift and phosphorylation in order to reduce cardiomyocyte stiffness. Local delivery of polylaminin may be a novel therapeutic strategy to reduce diastolic dysfunction and ameliorate the symptoms in HFpEF.

The paper of Pecorari et al. [9] describes the role of cytoskeletal cross-linkers in the onset and development of cardiomyopathies. Actinin alpha 2 (*ACTN2*), Filamin C (*FLNC*) and Dystrophin (*DMD*) have been described as causal genes in several cardiomyopathies. These genes encode for cytoskeleton proteins that not only have structural function but also regulate cell behavior through mechano-transduction. In cardiac myocytes, ACTN2 and FLNC localize to the Z-disc of the sarcomeres and interact with the intercalated disc (ID) components, while DMD localizes to the inner face of the plasma membrane. Z-disks, IDs and plasma membrane are important signaling hubs for cardiac myocytes. As ACTN2, FLNC and DMD, functioning as cross-linkers, interact with all these structures, mutations in these proteins are expected to have deleterious effects on cardiac myocytes structure and function, including abnormal activation of molecular pathways. Further mechanistic studies are needed to reveal the effects of mutations in these cytoskeleton elements on signaling pathways implicated in the onset and development of inherited cardiomyopathies. The findings could facilitate the development of novel therapeutic drugs to treat inherited cardiomyopathies.

The review by Gao et al. [1] focuses on molecular and cellular mechanisms in the pathogenesis of ACM. The review describes established ACM pathogenic mechanisms, such as activation of the Hippo pathway and of the TGF beta signaling and inhibition of the canonical Wnt signaling. Furthermore, the review reports recently proposed pathogenic mechanisms, including abnormal calcium homeostasis and inflammatory/autoimmune response. In addition, the review proposes a novel hypothesis based on the authors’ preliminary data about the role of epicardial cells and paracrine factors in the pathogenesis of ACM. The review underlines the complexity of the ACM phenotype, which must be considered the result of multiple cell–cell, cell–ECM cross-talks and molecular interactions. Finally, the authors discuss potential innovative therapeutic approaches based on the growing knowledge in the field.

In summary, the papers published in the present Special Issue describe the causal and the contributing factors of the phenotypic expression of non-ischemic cardiomyopathies, including genetic background, circulating microRNAs, paracrine factors, extracellular matrix modifications, cellular and molecular interactions and external factors such as lifestyle. Moreover, the published papers highlight the complexity of the pathogenesis of these diseases and the necessity of further studies in order to improve the diagnosis and allow the development of novel personalized treatments to prevent and/or attenuate the phenotype of patients with cardiomyopathies.
